# Mitigation of Mycotoxins in Food—Is It Possible?

**DOI:** 10.3390/foods13071112

**Published:** 2024-04-05

**Authors:** Eliana Badiale Furlong, Jaqueline Garda Buffon, Maristela Barnes Cerqueira, Larine Kupski

**Affiliations:** Laboratory of Mycotoxins and Food Science (LAMCA), School of Chemistry and Food, Federal University of Rio Grande, Av. Itália, km 8, s/n, Rio Grande 96203-900, Rio Grande do Sul, Brazil; dqmebf@furg.br (E.B.F.); jaquelinebuffon@furg.br (J.G.B.); maristelabarnes@furg.br (M.B.C.)

**Keywords:** antifungal agents, bioaccessibility, processing parameters, risk of exposure

## Abstract

Among microorganisms found in food, fungi stand out because they are adaptable and competitive in a large range of water activities, temperatures, pHs, humidities and substrate types. Besides sporulating, some species are toxigenic and produce toxic metabolites, mycotoxins, under adverse biotic and abiotic variables. Microorganisms are inactivated along the food chain, but mycotoxins have stable structures and remain in ready-to-eat food. The most prevalent mycotoxins in food, which are aflatoxins, fumonisins, ochratoxin A, patulin, tenuazonic acid, trichothecenes and zearalenone, have maximum tolerable limits (MTLs) defined as ppb and ppt by official organizations. The chronic and acute toxicities of mycotoxins and their stability are different in a chemical family. This critical review aims to discuss promising scientific research that successfully mitigated levels of mycotoxins and focus the results of our research group on this issue. It highlights the application of natural antifungal compounds, combinations of management, processing parameters and emergent technologies, and their role in reducing the levels and bioaccessibility. Despite good crop management and processing practices, total decontamination is almost impossible. Experimental evidence has shown that exposure to mycotoxins may be mitigated. However, multidisciplinary efforts need to be made to improve the applicability of successful techniques in the food supply chain to avoid mycotoxins’ impact on global food insecurity.

## 1. Introduction

Concern for food security has always been present in human civilization and the relation between food quality and health has always been clear in every culture. Science and technology evolution has allowed the definition of policies on food security with solid fundamentals [1]. However, throughout history, the risk of global food insecurity has been increased by many factors, such as inadequate economic distribution, low availability of commodities and climate changes [2,3]. The climate is an important issue to ensure food security because it affects the quality and quantity of food production; for example, the effects of the climate on profiles of fungi and mycotoxins are challenges to ensure the availability of food safety [4,5,6,7].

The consequences of fungal contamination in food are almost unavoidable because it may occur in any stage of the supply chain, from the field to ready-to-eat food [5,7]. Besides their ability to sporulate, fungi easily adapt to several biotic and abiotic conditions [6]. Toxigenic fungal species randomly release toxic secondary metabolites called mycotoxins, which remain in the food matrix even if the microorganism has been inactivated [8]. In general, mycotoxin production is related to adverse biotic and abiotic conditions in the substrate or environment [5]. The main producers of mycotoxins are species that belong to the genera *Aspergillus*, *Alternaria*, *Fusarium* and *Penicillium*. The most prevalent mycotoxin families—aflatoxins, fumonisins, ochratoxin A, patulin, tenuazonic acid, trichothecenes and zearalenone—in food have maximum tolerable limits (MTLs), defined as ppb or ppt, in many countries to prevent health damage. Not only their chemical stability but also their chronic and acute toxicity vary in families and in food matrixes, a fact which requires specific tools to mitigate the effects on animal and human health [9,10,11].

The damage caused by the consumption of food contaminated with fungi has been known for a long time, but its scientific basis increased around the 1960s [8,9]. The topics and trends of mycotoxicological problems addressed in the last 20 years were found on scientific search platforms, such as Science Direct, Scopus and Medline. The average number of papers published on the topic was 120 per year, concentrated in the last ten years. The profile of the topics is summarized in Table 1.

Every topic mentioned in Table 1 is relevant and contributes to adopting strategies and protecting human and animal health. They also contributed to mitigating the risk of exposure to mycotoxins. However, specifically, studies of mitigation are restricted to 10% of the experiments. Therefore, some gaps related to unavoidable critical issues remain [9]. Challenges of mycotoxin mitigation are the random occurrence of fungi and their derivates as trace concentrations whose diverse chemical structures, toxicity and stability in the same chemical family make it difficult to model or avoid their distribution in food supply chains. Additionally, acute and chronic symptoms of exposure are not specific, a fact that makes it difficult to identify the cause and treat the damage. Therefore, agro-industrial losses related to fungal contamination are a global cause of food insecurity [9,10].

To mitigate dangerous factors means decreasing their impact on human and animal health and on the environment. The term mitigation applied to mycotoxins means taking actions to minimize their toxic effect on consumers’ health [12,13,14]. It consists either of using tools to prevent fungal growth in a substrate or avoiding mycotoxin release in it [13,14,15,16,17]. If the matrix is contaminated, to mitigate mycotoxins, it is necessary to alter their chemical structures by physical (thermal, extrusion, UV light, ultrasound, electromagnetic field, plasma treatment), chemical (addition of ammonia, SO_2_, acid, alkaline and oxidant reagents) and biological techniques (enzymes or microorganisms) that degrade, break or reduce their toxic forms to limit their ingestion [11,13,18]. It should be highlighted that physical and chemical treatments usually require more drastic parameters to cause mycotoxin degradation and may result in dangerous chemical residues which decrease food quality [18,19].

The term decontamination is suitable to refer to techniques that allow separation or isolation of toxic compounds from their matrix. Sorting, sieving, floating, washing, dehulling and milling degrees have been successful in promoting a reduction in exposure to mycotoxins [12,13]. However, their efficiency is limited in some matrices because the physical characteristics of the material define the processing steps and, along the food chain, other fungi may contaminate them and release mycotoxins [8,18]. A reduction in toxicity is better proved when it is shown, at least, by a reduction in mycotoxins’ bioaccessibility, rather than by a mere decrease in their levels detected in food [9,11]. The application of mitigation strategies requires evaluation of mycotoxins’ bioaccessibility because they interact with the food matrix or have masked forms, making it difficult to determine their actual level in food by conventional analytical procedures. The determination of mycotoxins’ bioaccessibility consists of simulated gastric digestion followed by quantification of their level in the digested material [10,20].

Overlapping of both terms, the role of strategies and the questionable efficiency of actions to mitigate the damage result from the fact that more than one may be applied along the food chain and cause controversial results in the same family of mycotoxins. This scenario leads to the question: “Is it possible to find efficient strategies to mitigate the effects of mycotoxicological contamination by applying multidisciplinary knowledge and tools?” To answer the question, the knowledge produced by different studies on the topic must be collected and the contribution of these results must be critically evaluated in the context of mycotoxin mitigation.

This critical review aims to discuss promising scientific research that successfully mitigated levels of mycotoxins and to focus on the results of our research group. It highlights the application of efficient natural anti-fungal compounds, combinations of management, processing parameters and emergent technologies, and their role in reducing the detected levels and bioaccessibility. We hope that these promising experimental tools should motivate experts whose multidisciplinary efforts are able to effectively reduce the impact of mycotoxin exposure as a factor in global food insecurity.

## 2. Preventive Action

### 2.1. Policies to Reduce Exposure to Mycotoxins

Based on scientific data issued by national and international organizations, such as the National Health Surveillance Agency, Ministry of Agriculture and Livestock in Brazil, Food and Drug Administration, Food and Agricultural Organization, Codex Alimentarius and European Community, groups of academic and independent researchers have proposed tolerable limits and management protocols to reduce the risk of exposure to mycotoxins by food ingestion. Everyone agrees that the first step is that laboratories, which use validated methods and a system of quality control, should continuously monitor and issue reliable data to ensure effective application of tolerable limits of contaminants in food. Professional qualification, which is also fundamental to reaching this target, has been successful in different regions.

Many countries have their MTLs in the range of ppb or ppt for mycotoxins, such as aflatoxins, fumonisins, ochratoxins, patulin, trichothecenes and zearalenone, in corn, wheat, oat, milk, coffee, rice and their products. MTLs are determined mainly by the toxicity, occurrence and frequency of the contaminant in human and animal diets. Their compliance is evaluated by official organizations and commercial trading. However, this procedure is not applied worldwide [9].

### 2.2. Preventive Actions Pre- and Post-Harvest

Tools to select plant species that were more resistant to pests, the climate and other abiotic factors were first used for preventing losses in crop productivity [16,21]. Later, they were used for preventing fungus contamination, selecting taller plant varieties richer in indicators of defense mechanisms (enzyme inhibitors and phenolic compounds) and enabling good adaptability to adverse environments. Advances in molecular techniques have improved genetic modifications that have resulted in plants that are more resistant to fungal attack [11,18].

Official protocols of crop management of the most important commodities recommend soil preparation, crop rotation, the use of pesticides (fungicides) and pest biological control [22,23,24]. *Bacillus subtilis* [25] and *Trichoderma afroharzianum* [26] have been efficient in decreasing fungal contamination in fields. To avoid fungal contamination during storage, drying at suitable temperatures, followed by aeration, purging and sanitation of the warehouse, is recommended [10,18]. However, climate change has challenged the efficiency of these practices because it has affected the geographic distribution of fungi and their toxicity [4,7].

Even though fungicides, the most common tool, inhibit fungal growth, they leave residues that are dangerous to health, while some of them act as stressing factors and cause manifestations of toxigenic potential. Badiale Furlong et al. [27] reported that Heidtmann-Bemvenuti et al. [28] showed that strobilurins and tebuconazole were related to the highest deoxynivalenol (DON) level in rice products, while other authors reported similar behavior in wheat, corn and rye [29,30,31]. These facts have motivated studies of natural compounds which have shown antifungal properties in crops and food supply chains to protect against mycotoxicological losses [29,32,33].

**Figure 1:**
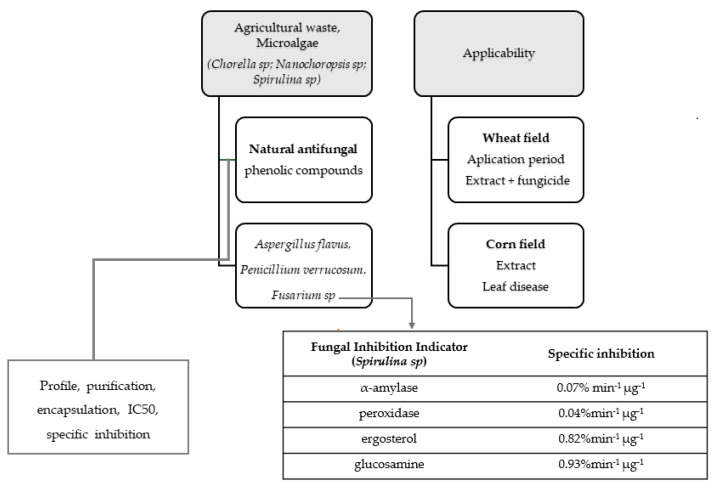
Antimycotoxigenic effects of microalgae extracts.

Regarding natural antifungal compounds, many promising results may be found in the literature. The inhibitory fungal potential of spices, essential oils, phenolic compounds from different sources, oryzanol, curcumin and other plant extracts has already been demonstrated [7,18,29,30,34,35,36,37,38]. Compounds which inhibit fungal growth or mycotoxins releasing act on the cell wall or cell membrane integrity, enzyme activities, genic expression and the respiratory chain. It should be highlighted that there is a thin line between their levels of benefits and stress to fungal cells. The antifungal and antimycotoxigenic activities of plant phenolic extracts were shown by [27], with α amylase inhibition resulting from protein extracts from rice, oat and wheat, evidenced by Pagnussatt et al. [39] and Pagnussatt et al. [40], and inhibition of gen Tri5 of *Fusarium graminearum* by rice phenolic extracts, evidenced by Heidtmann-Bemvenuti, Tralamazza, Jorge Ferreira, Corrêa and Badiale-Furlong [28]. Although many studies have described the antifungal effects of phenolic extracts from different sources, there are few demonstrations of applications to food production. Figure 1 shows a diagram of a promising study that demonstrated the applicability of phenolic extracts from microalgae as natural fungicides. It highlights the steps from extraction to field application to prevent fungal contamination in wheat and corn food chains.

Assays that show the antifungal effects of microalga phenolics extracts began by establishing a protocol to extract them from microalgae. Extracts from *Chlorella spp* and *Spirulina platensis* were able to decrease 53% of *Aspergillus flavus* growth by inhibiting the synthesis of cell membranes [41,42]. The antifungal activities of microalga ethanolic extracts (free and encapsulated) were also evaluated against 12 *Fusarium graminerum* strains and trichothecene production [27,43,44,45]. The authors evaluated the effects of extracts and determined inhibition of halo development, specific inhibition of α amylase and peroxidase activities and production of ergosterol and glucosamine, in comparison with untreated fungal cultures. Encapsulation of phenolic extracts in phosphatidylcholine improved their ability to avoid fungal growth in culture media 1.5-fold; this was shown by a decrease in the halo diameter and by glucosamine and ergosterol production. The percentages of decrease in DON, nivalenol and 15 acetyl deoxynivalenol production were 98%, 89% and 77%, respectively [43]. Despite the efficiency of encapsulation for fungicide activity, it is expensive in large-scale applications. Purification of phenolic extracts from *Nannochloropsis* sp. and *Spirulina* sp. did not show significant inhibition of fungal growth [27]. This was a promising result because it reinforced that a high degree of purification was not necessary because the synergic activity of phenolic acids is needed for efficient fungal inhibition. This makes their application to food supply chains more sustainable.

The applicability of phenolic extracts from microalgae to experimental wheat and corn fields was evaluated by Scaglioni, Scarpino, Marinaccio, Vanara, Furlong and Blandino [44] and Scaglioni et al. [46]. The effects on the severity of fusariosis were evaluated on experimental fields after applying phenolic extracts, their mix with a synthetic fungicide, positive control (tebuconazole) and negative control (without any natural or synthetic fungicide) at different growth stages of plants. In the wheat field alone, phenolic extracts inhibited DON production (58%), while the mix of phenolic extracts and fungicide inhibited 38% [44]. Foliar disease and fumonisin concentrations were reduced by applying phenolic extracts mixed with tebuconazole [46]. Regarding post-harvest effects of natural antifungals, there is evidence that phenolic extracts from agro-industrial residues fermented by *Rhizopus oryzae* were able to avoid fungal spoilage and mycotoxins in bakery products [47] and to inhibit production of trichothecenes and emerging mycotoxins in corn [48].

Even though there is evidence of beneficial effects of natural fungicides from plants or microorganisms on the prevention of fungal growth and mycotoxin production, there are still many gaps to make their application become routine in food supply chains because they are secondary defense metabolites in their sources. Therefore, resulting quantities are not enough to meet high demands and some active compounds are inactivated during extraction or application parameters. To produce a mix of phenolic compounds by chemical or biological synthesis which is similar to microalga extracts should be a great solution for the future of agrobusiness and food security.

### 2.3. Degradation of Mycotoxins

To degrade mycotoxins means inactivating, destroying or removing groups responsible for toxicity from their structure, neither producing toxic residues nor affecting the nutritional values and technological properties of food [13,15,49]. Physical tools may change the native structural performance of mycotoxins or their interaction with chemical components found in food matrices. They may be dry or wet heat treatment, extrusion and application of UV light, δ radiation or plasma to the contaminated material, under different parameters, mainly regarding their magnitude and time of exposure [12,50,51,52]. Chemical techniques use acid, alkaline, reducing and oxidizing reagents which cause hydrolysis, ammoniating, oxidation and sulfonation in parts of structures that are related to toxic effects on consumers [18,53,54,55,56]. Biological techniques are those that use enzymes or microorganisms whose activities modify groups in the structure or destroy mycotoxins [49,57,58,59,60]. Along the food chain, more than one of these techniques may be applied to a certain matrix. Applicability to and efficiency of mycotoxin degradation are affected by their structural susceptibility to environmental parameters, matrix composition, nutritional and technological effects, and the formation of unknown third compounds [9,12,18,61]. In this context, the term degradation is related to treatments that cause great and beneficial alteration (toxicity reduction) to mycotoxin structures.

#### 2.3.1. Degradation of Aflatoxins

**Figure 2:**
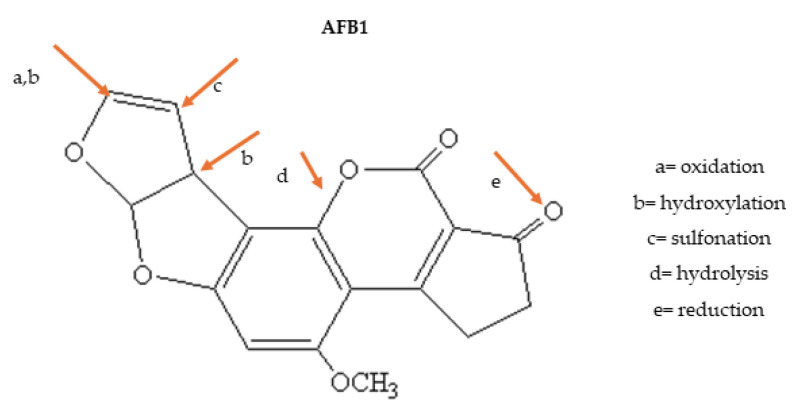
Critical points to reduce aflatoxin B1 toxicity.

Aflatoxins are mycotoxins which have been assessed by different studies because they are frequently found in several food matrices worldwide. Their chemical forms have different levels of toxicity whose effects are shown by gastrointestinal damage, reduction in immunological defense and carcinogenicity. In this family, the main aflatoxins in food are B1, B2, G1, G2, M1 and M2, whose chemical structures differ in some points around cyclic groups (Figure 2). They determine their stability and toxicity. Health damage by aflatoxins has been prevented in many countries by establishing MTLs that are complied with in commercial operations. Aflatoxins are mainly produced by *Aspergillus flavus* and other species of this genus whose challenge to avoid contamination is their thermal resistance and relatively high temperatures for mycotoxin production (25–28 °C) [62].

The main points of the structure of aflatoxins that should be changed by several strategies to reduce their toxicity are shown in Figure 2. The aflatoxin B1 (AFB1) structure is marked with orange arrows and letters around the cyclic groups whose changes may reduce their dangerous effect on human and animal metabolism. Since aflatoxins have similar structures, it may be inferred that changes in the highlighted points may also mitigate the toxicity of other aflatoxin forms.

Changes in aflatoxin B1 structure may be caused by oxidation, hydroxylation, sulfonation, hydrolysis and reduction in positions a, b, c, d and e. Physical, chemical, microbial and enzymatic actions and their combination have been studied [62,63]. Almost every change in AFB1 may cause a 3–10-fold decrease in toxicity and their derivative forms have been found in food matrices as results of natural transformation or processing parameters. However, oxidation of the double bond of the furanic ring (in the left, highlighted as a, b) and release of the epoxide ring are dangerous. In this form, the structure easily crosses nuclear membranes and reacts with nitrogen bases of nucleic acid, resulting in DNA adducts related to the carcinogenicity of AFB1. It should be highlighted that epoxidation may also occur during detoxification reactions in human and animal metabolism [8,12,54]; this explains why the liver is the preferential target of aflatoxins. The fate of aflatoxins in food supply chains and in the environment is influenced not only by biotic and abiotic parameters of field management, storage, processing and domestic food preparation but also by the solubility and structural stability of mycotoxins [62,64].

Studies of aflatoxin mitigation have investigated the supply chains of corn, peanuts, rice, cocoa, coffee, milk and nuts. Considering the importance of these food chains, there are certain treatments whose benefits are consolidated and have been applied at the industrial scale [51,54]. Some examples are wet milling, which reduced 67% of initial contamination in corn [65]; hydrothermal treatment, which decreased 57% of the initial level of rice contamination [66,67]; δ irradiation, which was applied to coffee and cocoa and showed controversial results [12]; alkaline treatment applied to feed, which can reduce the nutritional value of food matrices [56]; sulfite reduction, whose application is restricted [8]; and solid and liquid fermentation with fungi and bacteria [49,63,68].

There are promising and appliable results of conventional thermal [69] and emergent technologies, such as microwave heating [66], extrusion [69], enzyme degradation of AFB1 and M1 in milk [68], gamma and electron beam irradiation, ultraviolet and pulsed light, electrolyzed water and cold plasma [56,63]. However, in general, degradation levels are below 100%. The applicability of these tools to degrade mycotoxins is not common in industrial sites [70]. Consequently, they should be carefully studied before being used for mitigating exposure to aflatoxins.

#### 2.3.2. Degradation of Ochratoxins

**Figure 3:**
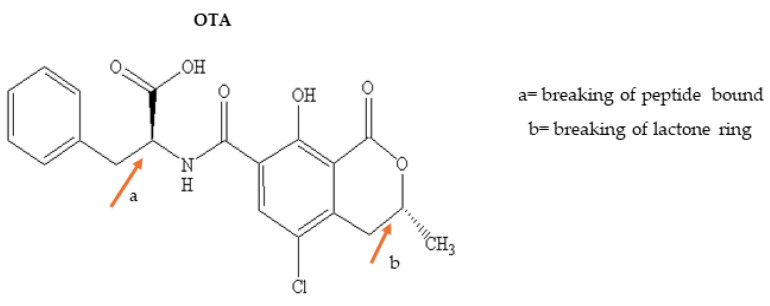
Critical points to reduce OTA toxicity.

Ochratoxins comprise a group of mycotoxins produced by fungi that belong to the species *Aspergillus ochraceous*, *Penicillium verrucosum* and others. They are derivatives of phenylpropane metabolism as a defense against adverse abiotic and biotic parameters [71,72]. Ochratoxin A (OTA) is related to nephrotoxic and hepatotoxic damage; thus, the toxicity, frequency and evidence of its damage to human and animals led to the establishment of MTLs in food products based on cereal and fruit [13,73]. OTA consists of a coumaric group bound by peptide linkage to phenylalanine (Figure 3). Its toxicity may be reduced by changes in its structure in the highlighted position in Figure 3, i.e., hydrolysis of the peptide linkage and opening of the lactone ring.

The breaking of peptide bonds releases ochratoxin α, which is 5–10-fold less toxic than OTA. OTA and its derivatives has been detected in cereal, the grape, cocoa, and coffee. Degradation or transformation of the chemical structure of OTA may also release “modified ochratoxins”, such as ochratoxin B (OTB), ochratoxin C (OTC), OTα, ochratoxin β (OTβ), OTA methyl ester (MeOTA), OTB methyl ester (MeOTB), OTB ethyl ester (EtOTB), OTα methyl ester, OTA ethyl amide and OTA glucose ester [13]. They may be produced by fungal species as a result of substrate composition or as a response to a treatment. For example, changes in pH favor interconversion among OTA derivatives [12,71]. There is evidence that carboxypeptidase A [74] and peroxidase are able to reduce OTA levels detected in wheat bran and meal after carboxypeptidase action and fruit juice and beer after peroxidase application [59,74,75]. The advantage is that enzyme specificity and activity under mild conditions prevents alteration to other compounds in matrices. Other physical (adsorption by magnetic silver and copper nanoparticle) and chemical treatments (feed ammoniation and alkaline treatment of corn and cocoa) have had their potential to reduce OTA levels in laboratory studies. The reduction ranged from 10 to 95% [75] and data showed that enzyme and hydrothermal treatments are the most efficient ones [74]. Dry thermal treatment was efficient at reducing (57%) OTA levels detected in coffee, but the temperature needs to be high; a fact that increases the risk of hiding processing contaminants. The fermentation step of cocoa processing is promising to reduce around 30% of detected OTA levels in comparison with initial contamination [12,13].

In addition to its toxicity, OTA may decrease wine functionality, a fact that motivated a review of OTA from grapes to wine and highlighted that red wines have higher OTA levels than white ones and that throughout winemaking they tend to decrease, except during maceration [57,76]. Some adsorbents, such as gelatin, egg albumin and bentonite, may mitigate OTA levels but cannot degrade them, just like some yeast species that also bind mycotoxins [56,60].

Despite several reports of mitigation of OTA levels by certain treatments, they are restricted to laboratory studies and information about bioaccessibility and bioavailability is still scarce [9].

#### 2.3.3. Degradation of Trichothecenes

**Figure 4:**
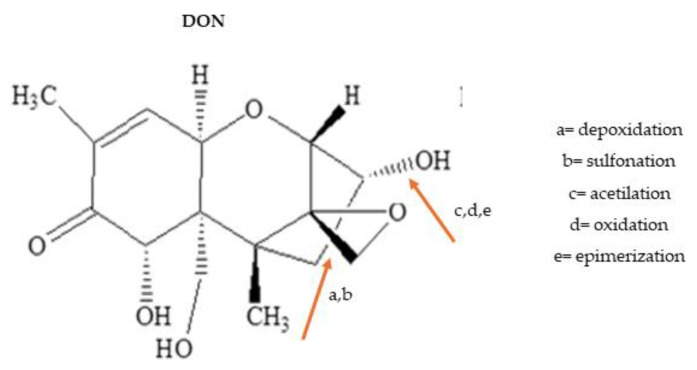
Critical points to reduce DON toxicity.

Trichothecenes comprise a group of mycotoxins synthesized by fungi that belong to the genera *Fusarium*, *Myrothecium*, *Trichoderma*, *Trichothecium*, *Cephalosporium*, *Verticimonosporium* and *Stachybotrys* from polyketides whose cyclization results in a trichothecene ring with a 12–13 epoxide ring, which is mainly responsible for the family’s toxicity (Figure 4) [77]. They are classified as A, B, C and D trichothecenes, depending on groups linked to the basic structure of trichothecene rings. Trichothecenes A and B contaminate cereals and other substrates that are rich in carbohydrates and exhibit high water activity that allows fungal growth. Their toxicity is a consequence of the effect on different steps of protein synthesis, from translation to elongation of the protein chain [78]. The main symptoms of exposure are gastrointestinal disorders, depression of the immunological system and damage to neurological functions [79].

Since the identification of the risk of exposure to group B trichothecenes, mitigation strategies have been mainly implemented in the processing chain of wheat, corn, oat, rice and rye [79]. The most studied trichothecene belongs to group B, DON, which has MTLs defined in many countries and commercial trading [78]. Since the 1990’s, when its acetylated and glycosylated derivatives were detected in cereal-based food, their interconversions in the processing chain and in digestive reactions have also been a concern [78]. Because of their frequency and toxicity, many physical (thermal treatment, exposure to different regions of electromagnetic spectra, cold plasma irradiation), chemical (acetylation, ammonia, alkaline treatment) and biological (enzyme and microorganism degradation) strategies and their mixes have been studied to degrade and detoxify trichothecenes, mainly DON [61,77,80,81,82,83]. Some of them were successful but none have reached total contaminant remotion and few have been adopted at the industrial scale because they may affect the nutritional and technological properties of products; additionally, their applicability to the industrial environment is not always sustainable [9,84,85].

Figure 4 shows DON’s structure and reactions in points which are critical to reduce its toxicity by physical, chemical and biological parameters applied along the food supply chain [12,78].

A decrease in detected DON levels or its derivatives and/or change in their proportions is a consequence of deepoxidation, sulfonation, acetylation, oxidation and epimerization reactions, which depend on responses given by fungi to the environment or to substrate composition [78] and on the chemical composition of formulations or the addition of chemical reagents, which are not allowed in many countries [77]. The combination of thermal and biological parameters in bakery and beverage production mitigates DON in ready-to-eat food. Malting and fermentation reduce 78% of initial contamination levels. Alkaline treatments lead to a 36% decrease in DON levels in corn [78]. There are studies of degradation of group A trichothecenes (toxin T2 and HT2) by alkaline, acid and enzyme hydrolysis [12,17,61,77,80,82,84,85].

Garda-Buffon and Badiale-Furlong [86] showed the kinetics of DON degradation during submerged fermentation by *Aspergillus oryzae* and *Rhizopus oryzae* and highlighted the relation between low levels of DON and peroxidase activity in submerged fermentation. The activity of DON during baking has been studied and there is evidence that it is reduced during baking but that it remains in the solid residue after alcoholic fermentation with *Saccharomyces cerevisae* [78,84,85,87]. It should be emphasized that a reduction in detected levels does not mean a reduction in risk exposure, because toxic residues may remain in food products and, during digestion, DON may be release from masked forms, absorbed and metabolized [78,84,85]. This fact justifies the trend to determine risk based on the estimate of trichothecene bioaccessibility [9,88].

#### 2.3.4. Degradation of Fumonisins

**Figure 5:**
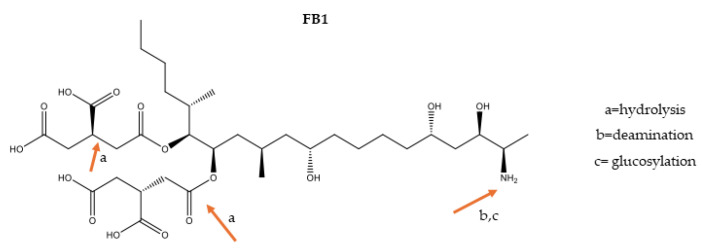
Critical points to reduce FB1 toxicity.

Fumonisins comprise another group of Fusarium toxins produced by *Fusarium verticilioides*, *F. proliferatum* and *Aspergillus niger* which use the polyketide metabolism by elongation of the carbon chain and esterification with tricarboxylic acid in one end of the C chain and addition of an amino group to the other end of the C chain, resulting in a structure which is similar to phospholipids (Figure 5). Exposure to them inhibits sphingolipid biosynthesis and affects the neurological system, lung and brain. Evidence of carcinogenicity has also been reported. Fumonisins B1, B2 and B3, which differ due to the absence or presence of hydroxyl groups, have been found in cereals, such as corn, and grapes. Their toxigenicity determines the establishment of health-based guidance values for the sum of three 2 μg/kg body weight/day in many countries [89], aligned with the SCF recommendation (2003).

Since corn and its products are frequently contaminated, they are the most studied commodity regarding the impact of fumonisins. Concerning their mitigation, many reports focus on the corn supply chain and evaluate physical, chemical and biological strategies, whose results have been related to characteristics of food and processing parameters. Even though promising tools have been found, few have been applied on a large scale [11,20]. A reduction in the toxicity of fumonisins—and other mycotoxins—depends on the degradation of their chemical structure. Figure 5 shows fumonisin B1’s (FB1) structure and its main points which are critical to reduce toxicity by applying physical, chemical and biological treatments [12,18,90,91].

Hydrolysis of ester bonds may occur in alkaline and acid media, but it is not always suitable for nutritional properties. The use of acid preservatives should be beneficial to reduce fumonisin toxicity because it also hydrolyzes ester bonds, but the efficiency is limited by the reverse reaction. Esterase enzymes from microorganisms, such as the yeast *Exophiala spinifera* and bacterium *Sphingomonas* spp., catalyze hydrolysis of ester bonds in the polyketide chain and may be useful to decontaminate corn products [13,14,88].

Massarolo, Rodrigues, Ferreira, Kupski and Badiale-Furlong [65], Massarolo, Ferreira, Collazzo, Bianchini, Kupski and Badiale-Furlong [69] studied the effects of parameters of corn processing and preparation on FB1 levels and bioacessibility. The authors found that, after dry milling, 23% of mycotoxins remains in the endosperm. Hydrothermal cooking reduced initial contamination from 39 to 59%, depending on the addition of salt, lipid and water ratios; a combination of parameters in the extrusion process reduced the initial content of FB1 from 65 to 79%. The authors reported that FB1 decreased due to its interaction with resistant starch in the hydrothermal treatment of cornmeal. A combination of composition and treatment parameters explained the level detected in the ready-to-eat product, but after the digestion process the level was higher than before, i.e., it increased from 73 to 85%. Laboratory studies showed that the use of esterase enzymes, fermentation to produce alcoholic beverages and magnetic nanoparticles as adsorbents led to an 86% decrease in the initial contamination.

This fact reinforces the importance of evaluating the bioacessibility of contaminants after every tool is applied to reduce the risk of exposure and recommends the scale-up of the techniques as secure parameters to mitigate contamination by fumonisins.

### 2.4. Decontamination of Mycotoxins

The term decontamination is suitable to refer to elimination of toxic compounds found in a food matrix. Therefore, knowledge of the occurrence resulting from reliable methods, characteristic of fungal colonization and processing steps, is the basis for the implementation of protocols to reduce or eliminate mycotoxins along the food supply chain [1]. Even though some tools play a key role in ensuring safe food, their application is limited [8,92].

Since fungi are aerobic, and they are found on the external portion of the matrix [10]. Thus, removing and washing cereal shells and fruit skins are successful and easy ways that may be used along the chain. Removing external portions of grains and fruit by sorting, sieving, floating, washing, dehulling, steeping and milling have proven to be efficient at decreasing levels of trichothecenes, aflatoxins, ergot alkaloids, fumonisins and zearalenone in cereals, patulin in apples, ochratoxin A in grapes, cocoa and coffee beans, aflatoxins in cereals, cocoa and coffee beans and adsorption by inert material of aflatoxins, ochratoxin A and fumonisins in corn [18]. The decontamination levels promoted by these protocols ranged from 6 to 58%, on average [11,16,24,93]. It should be highlighted that these tools have been adopted as good practices in food industries and in households [9]. When contamination is apparent, and shells and skins are structures that are easily isolated from edible portions, sorting and sieving tools are efficient at reducing mycotoxin levels from 12 to 73%. Dry and wet milling may be applied to grain processing, but its efficiency is affected by the solubility of mycotoxins in water, which may range between 22 and 47%, in comparison with the initial level. Adsorption by inert material is useful to apply to feed despite the risk of decreasing its nutritional value; some reports have shown that mycotoxins may decrease from 18 to 48% [12,15,51].

Considering previously mentioned strategies to reduce mycotoxin levels, their efficiency is also determined by the morphological characteristics of the material, in addition to the chemical properties of mycotoxins, such as their solubility and interaction with matrix components [51].

Dors et al. [94] and Prietto et al. [95] showed that, in rice processing, 45% of aflatoxins remain in bran and that, in rice parboilization, 63% migrate to the endosperm. Massarolo, Rodrigues, Ferreira, Kupski and Badiale-Furlong [65] studied the fate of aflatoxin and fumonisin in corn milling and found that, in dry milling, 74% and 23% of aflatoxins and fumonisins, respectively, remain in the endosperm. In comparison with wet milling of corn, the behavior of these mycotoxins was inverse.

The formulation of food with the addition of fiber and polyphenols has been proven to be good at reducing mycotoxin levels that act by interacting among polyphenolic hydroxyl groups and mycotoxins and adsorption by van der Walls force with fiber and resistant starch [20,32,96]. Therefore, a combination of processing parameters that promote interactions among mycotoxins and macromolecules of the food matrix which would be irreversible in the digestive step should enable us to reduce exposure to trichothecenes [78,84,97], aflatoxins [66,69,95,98], fumonisin B1 [69,90] and other mycotoxins [13,18].

## 3. Final Remarks

The availability of fast, sensitive and green analytical methods has allowed for the continuous detection of fungi and mycotoxins in foods worldwide. Knowledge about mycotoxin toxicity at the molecular level has also improved. These facts reinforce concern for mycotoxins and the effects of climatic scenarios on their profile. Efficient management practices and quality control are applied to the food chain to decrease contaminants. However, depending on the initial contamination, matrix components and morphological and physical characteristics of the material, residual levels of mycotoxins may exceed MTLs and require innovative mitigation strategies.

Despite the challenges of fungal adaptability, the climate scenario and structural stability of mycotoxins, there are several experimental studies regarding the benefits of friendly prevention using natural fungicides, emergent techniques and a combination of processing parameters to promote mitigation of mycotoxins. To implement these protocols and establish global policies to mitigate the damage, experts in different areas should deeply evaluate mathematical tools to model fungal behavior, sensitive and specific analytical methods, emergent green processes and multidisciplinary assays. In this way, the role of mycotoxicological factors may be weakened regarding risks of global food insecurity.

## Figures and Tables

**Table 1:** Profile of topics found in scientific papers that address mycotoxicological issues (2000–2022).

Topics	Specific Trends	Percentage (%)
Damage × Health	Animal (pigs and poultry)	38 ^1^
Methods/relations with biotic and abiotic varieties	Trichothecenes (DON and derivatives), fumonisins, aflatoxins, masked mycotoxins	18
Mechanisms of action	Cytotoxicity (DON), receptors (FB_1_)	8
Contaminated food	Cereal-based products, fruit, milk and derivatives, beer, wine, chestnut and spices	20
Innovation	Experimental models, cell culture, enzymes and molecular technologies	6
Applied sciences	Biomarkers, epidemiology, mitigation of contaminants	10

^1^ Estimate of percentage considering papers that address mycotoxins in scientific sites.

## Data Availability

No new data were created or analyzed in this study. Data sharing is not applicable to this article.
